# Primary isolated tuberculous colitis of the ascending colon

**DOI:** 10.1590/0037-8682-0012-2026

**Published:** 2026-04-10

**Authors:** Elif Gündoğdu

**Affiliations:** 1 Eskişehir Osmangazi University, Faculty of Medicine, Department of Radiology, Eskişehir, Turkey.

A 67-year-old man presented to an outside hospital with abdominal pain, distension, and generalized fatigue. Laboratory evaluation revealed a positive fecal occult blood test, and the patient was referred to our institution with preliminary differential diagnoses of colorectal malignancy and inflammatory bowel disease. On admission, symptoms persisted, and vital signs were within normal limits. Erythrocyte sedimentation rate and C-reactive protein levels were elevated (42 mm/h and 20.6 mg/L, respectively), while other laboratory parameters and tumor markers were within normal limits. Computed tomography (CT) demonstrated diffuse and nodular thickening of the ascending colon wall, with pericolonic fat stranding and regional lymphadenopathy (Figure 1). Chest CT showed no evidence of pulmonary tuberculosis. Colonoscopy revealed five ulcerated lesions in the ascending colon, covered with exudate (Figure 2), along with luminal narrowing. Multiple biopsies were obtained. Histopathologic examination demonstrated severe chronic active colitis with necrotizing granulomatous structures and acid-fast bacilli. Polymerase chain reaction testing for *Mycobacterium tuberculosis* was also positive. Based on these findings, a diagnosis of primary tuberculous colitis was established, and standard four-drug anti-tuberculosis therapy was initiated. Primary tuberculous colitis is a rare extrapulmonary manifestation of tuberculosis, representing a small proportion of gastrointestinal tuberculosis cases, approximately 19% of which involve the ascending colon^1^. It remains a considerable diagnostic challenge because it may mimic inflammatory bowel disease, other infectious processes, and colon carcinoma owing to non-specific radiographic and colonoscopic findings. Therefore, histopathological confirmation is essential for accurate diagnosis^2^. 

**FİGURE 1:**
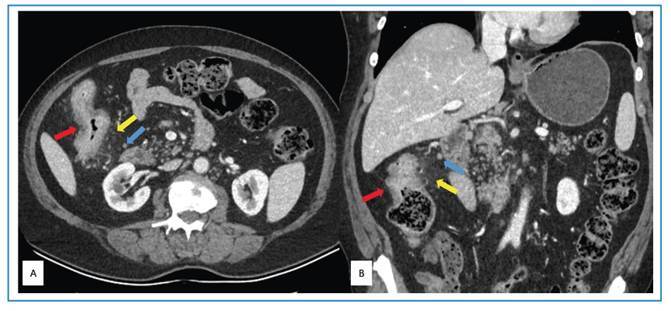
Axial **(A)** and coronal **(B)** computed tomography images showing thickening of the ascending colon wall (red arrows), pericolonic fat stranding (yellow arrows), and regional lymph nodes (blue arrows).

**FİGURE 2:**
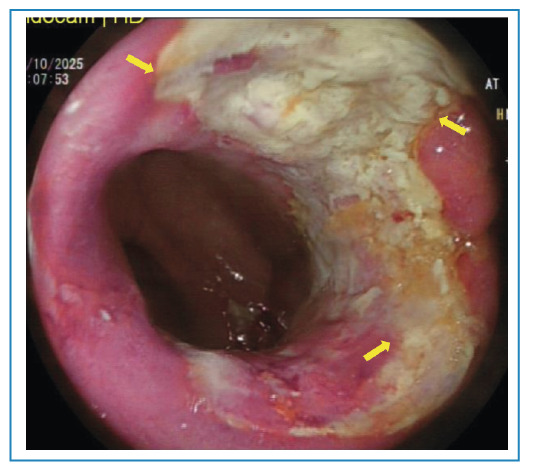
Colonoscopic image showing ulcerated lesions in the ascending colon covered with exudate.
